# Remote Programming in Patients With Parkinson's Disease After Deep Brain Stimulation: Safe, Effective, and Economical

**DOI:** 10.3389/fneur.2022.879250

**Published:** 2022-05-03

**Authors:** Pan Nie, Jibo Zhang, Xin Yang, Yuyang Shao, Xiuming Zhang, Wen Liu, Kai Fu, Jincao Chen, Jie Zhang

**Affiliations:** ^1^Department of Neurosurgery, Zhongnan Hospital of Wuhan University, Wuhan, China; ^2^Center for Functional Neurosurgery, Zhongnan Hospital of Wuhan University, Wuhan, China

**Keywords:** Parkinson's disease, deep brain stimulation, remote programming, telemedicine, equipment safety, cost

## Abstract

**Objective:**

The purpose of this study was to evaluate the safety, efficiency, and cost expenditure of remote programming in patients with Parkinson's disease (PD) after deep brain stimulation (DBS).

**Methods:**

A total of 74 patients who underwent DBS at the Department of Neurosurgery, Zhongnan Hospital of Wuhan University between June 2018 and June 2020 were enrolled in this study. There were 27 patients in the remote programming group and 47 patients in the outpatient programming group. Clinical data, programming efficiency, adverse events, expenditure, and satisfaction were compared between the two groups.

**Results:**

A total of 36 times of remote programming were performed on the 27 patients in the remote programming group, and four had mild adverse events during programming, and the adverse events disappeared within 1 week. The satisfaction questionnaire showed that 97.3% of the patients were satisfied with the surgical effect. The patients in the remote programming group (88.9%) were more likely to receive long-term programming after DBS than the patients in the outpatient programming group (74.5%). The Parkinsonism symptoms improved in both programming groups. The majority (18/27) of patients in the remote programming group lived away from the programming center, while the majority (27/47) of patients in the outpatient programming group lived in Wuhan, where the programming center was located (*P* = 0.046). The cost per patient per programming was US$ 43.5 in the remote programming group and $59.5 (56–82.7) in the outpatient programming group (*P* < 0.001). The median time cost for each visit was 30 min (25–30) in the remote programming group and 150 min (135–270.0) in the outpatient programming group (*P* < 0.001).

**Conclusion:**

Remote programming is safe and effective after DBS in patients with Parkinson's disease. Moreover, it reduces expenditure and time costs for patients and achieves high satisfaction, particularly for patients living far from programming centers.

## Introduction

Parkinson's disease (PD) is a chronic neurodegenerative disorder characterized by motor and non-motor disabilities (1). Deep brain stimulation (DBS) is currently an effective treatment for advanced Parkinson's disease. However, patients will face long-term and repeated professional care after surgery, which is closely related to the clinical effect of surgery (2, 3). There are many barriers to implementing professional programming and care in outpatient clinics, including geographic and financial constraints, and patient's ability to travel (4, 5).

Telemedicine, which can remotely provide healthcare services using telecommunication technology to provide medical services to patients living in remote areas, has been used for care and evaluation of patients with PD (6–9). Remote programming is a new application of telemedicine that allows patients to receive adjustments in medications and parameters at home. DBS stimulators (G102, G102R, and G102RZ; PINS Medical, Ltd., Beijing, China) with remote programming capabilities have been in use in China since 2017 and have been successfully applied in VNS postoperative remote programming (10). Parameters can be programmed *via* a remote programming platform, on which physicians and patients can communicate *via* video chat, and physicians can adjust DBS parameters *via* an internet and Bluetooth connection. However, there is currently no evidence on the effectiveness and safety of remote programming after DBS in patients with PD. Since 2017, we have been applying this system for the postoperative programming of patients with PD after DBS. In this study, we analyzed the clinical data, programming effect, adverse events, programming cost, and patient satisfaction of 74 patients with PD who underwent postoperative remote or outpatient programming in our hospital.

## Methods

### Patients and Grouping

Seventy-four patients who were implanted with a PINS DBS system (G102, G102R, or G102RZ) at the Department of Neurosurgery, Zhongnan Hospital of Wuhan University between June 2018 and June 2020 were enrolled in this study. The brain region targeted was the subthalamic nucleus (STN) of all patients. The diagnosis meets the diagnostic criteria of the British Parkinson's Society brain bank for primary PD (11). There were 27 patients in the remote programming group and 47 patients in the outpatient programming group according to the choice of patients and their families. The clinical data, programming effect, adverse events, programming cost, and patient satisfaction between the two groups were compared. The outpatient programming group was defined as each programming carried out in the outpatient department, and the remote programming group was defined as at least one programming performed through a remote programming system.

### Remote Programming Procedure

**Figure 1:**
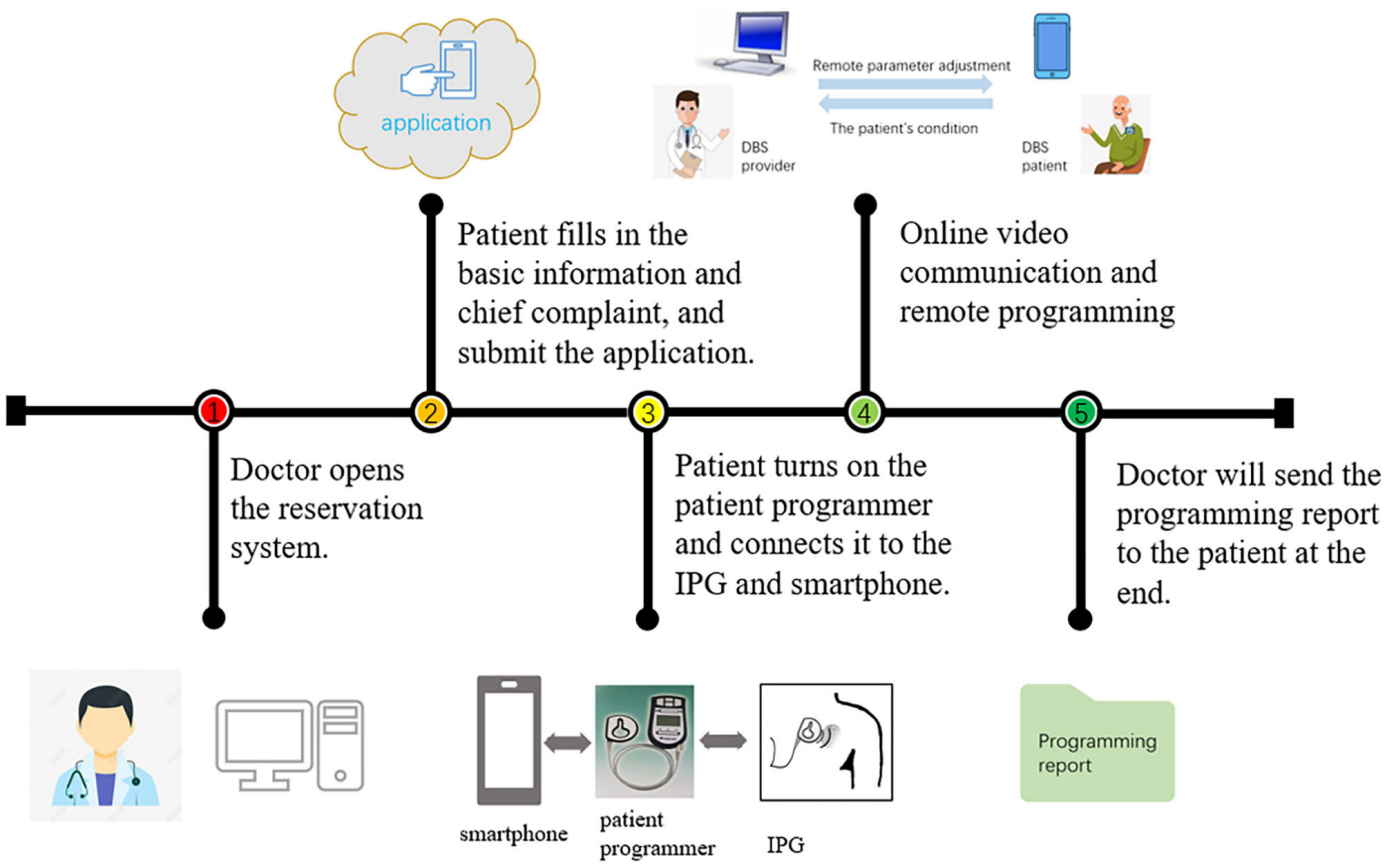
Flow chart for remote programming. IPG, implantable pulse generator.

**Figure 2:**
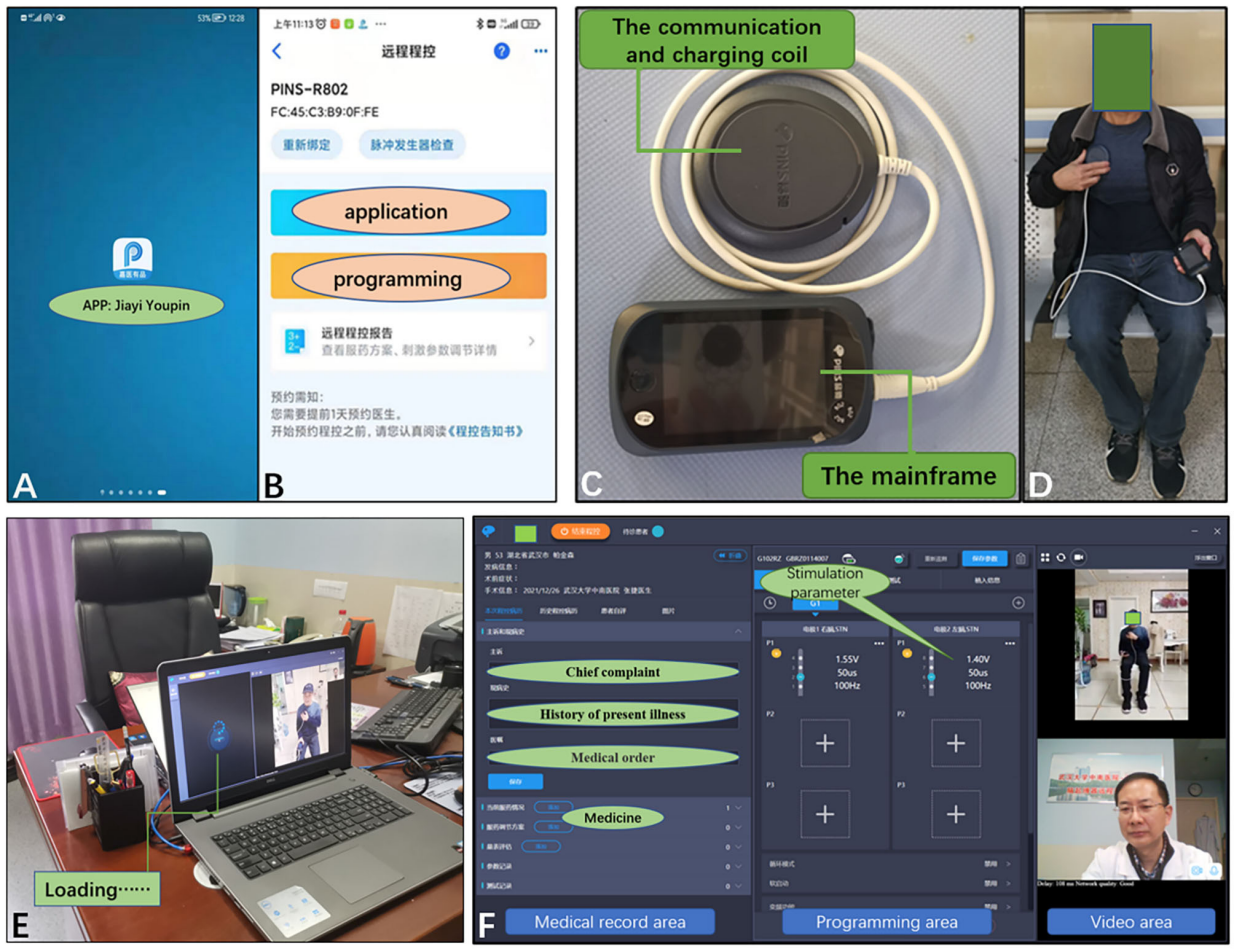
**(A)** Patients needed to download the programming app, PINS “JiayiYoupin” patient version. **(B)** Patients applied for programming using the app. **(C)** The patient programmer consisted of a communication/charging coil and a mainframe, which can be used for programming and charging the IPG. **(D)** Working status of the patient programmer. **(E)** Doctor was detecting IPG signals in patient. **(F)** Computer interface of the physician client during remote programming, as well as the display of its functions. IPG, implantable pulse generator.

The PINS remote programming system was adopted in this study. The remote programming center was located at Zhongnan Hospital of Wuhan University. The remote programming system mainly included a physician client (smartphone and computer), a patient client (smartphone), a patient programmer, and a server station (12). Before remote programming, the patients' family needed to download a PINS programming application (App, PINS “JiayiYoupin” patient version) to their smartphone and provide their personal information for verification. Through web service interfaces on the Internet, the server station built a virtual link between the physician client and the patient client. Programming procedure (Figure 1): (1) doctor would release programming permission in physician client (smartphone, PINS “JiayiYoupin” doctor version App); (2) patients should fill in the basic information and chief complaint and sign the remote programming agreement before they apply on the app, “JiayiYoupin” (Figures 2A,B); (3) a doctor would review and approve the application. During programming, patient's family should turn on the patient programmer, and keep the coil close to and connect with the implantable pulse generator (IPG) *via* near field wireless communication and connect it with the patient client through Bluetooth (Figures 2C–E); (4) the doctor communicated with the patient and their family members through the physician client and patient client by video chats on the Internet, and checked the patient's condition, and when the patient programmer was connected to the IPG, the doctor could check stimulation parameters, contact impedance, and battery voltage, and adjust stimulation parameters on the physician client under video monitoring, which included stimulation voltage, pulse width, frequency, and contacts, and, finally, observe a curative effect (Figure 2F); if necessary, the doctor could also adjust the amount of voltage, pulse width, and frequency on the patient programmer (Figure 2F); (5) after programming, the doctor would send the report to the patient client. If the network was interrupted during the adjustment, the system would automatically reset the last program parameters by default to avoid any harm to patients, and we would try to connect to the network again.

### Data Collection

Clinical data were collected, including gender, age at DBS implantation, course of disease, preoperative Hoehn-Yahr stage, preoperative Modified Unified Parkinson's Disease Rating Scale III (MUPDRS III, drug-off) without rigidity and pullback test scores (13), and preoperative Levodopa Equivalent Daily Dose (LEDD). Also, we collected and analyzed the MUPDRS III score at the last follow-up (drug-off, stimulation-on), LEDD, time costs of each programming, distance from the patient's residence to the hospital, programming-related adverse events, and cost of each visit in the two groups.

We calculated the costs and time spent by the patients and families on the programming or visit in both groups. In the outpatient programming group, the costs included traveling costs, outpatient service costs for each visit, and working day salary lost by the family for accompanying the patient to the hospital. The time of outpatient programming was spent on traveling and programming. In the remote programming group, there was no other expenditure except for the fee of RMB 300 (US$ 43.5) that was charged for the remote programming, and time was spent on programming only. RMB was converted to US dollar according to the annual average exchange rate in 2019. The last follow-up date of this study was June 30, 2020.

### Satisfaction Questionnaire

The scale of questionnaire was divided into two parts. The first part was about patients' satisfaction with the effect of DBS and willingness for long-term programming after surgery. The second part provided different questions for the two groups. The patients in the remote programming group were asked whether they were satisfied with the remote programming and reasons for satisfaction (open-type question). In the outpatient programming group, the patients were asked not only about their satisfaction with the outpatient programming but also their willingness for remote programming and reasons (open-type question).

### Statistical Analysis

The SPSS 22.0 software was used for the statistical analysis of the data. An Independent sample *t*-test was conducted for continuous variables with normal distribution. Measurement data not conforming to normal distribution were expressed as median and 25th and 75th percentiles, and the Wilcoxon rank-sum test was conducted for comparison between the two groups. Count data were expressed by the number of cases, and the comparison between the groups was performed by the χ^2^ test. *P*-value <0.05 was considered as a statistically significant difference.

### Ethics and Informed Consent

The study was approved by the Medical Ethics Committee of Zhongnan Hospital of Wuhan University. Informed consent was obtained from the patients and their families.

## Results

### Clinical Information of Patients

There were no significant differences in age, course of disease, preoperative LEDD and MUPDRS III score, and Hoehn-Yahr stage between the two groups. The MUPDRS III score and LEDD were improved at the last programming compared to that before the surgery in both groups, and there were no significant differences in improvements between the two groups. The distance from patients' residences to the hospital tended to be farther in the remote programming group. Further analysis of the residences of the two groups showed that most of the patients (18/27) in the remote programming group lived far away from Wuhan, and that most of the patients (27/47) in the outpatient programming group came from Wuhan where the hospital was located (*P* = 0.046, Table 1).

**Table 1:** Clinical data between remote and outpatient programming group.

	**Remote programming group *n* = 27**	**Outpatient** **Programming group** ***n*** **= 47**	***P***
Gender (M/F)	15/12	21/26	0.368^a^
Age (years)	60.77 ± 0.6	61.16 ± 0.6	0.810^b^
Course of disease (years)	9.24 ± 0.2	12.17 ± 0.3	0.061^b^
Hoehn-Yahr stage (X ± S)	3.20 ± 0.7	3.40 ± 0.8	0.387^b^
Pre-op. LEDD (mg)	688.0 (450.0–825.0)	600.0 (400.0–831.0)	0.363^c^
Decrease rate of LEDD (%)^d^	46.0 (31.0–58.0)	33.0 (8.0–57.0)	0.368^c^
Pre-op. MUPDRS III (medicine-off)	29.0 (24.0–35.0)	32.0 (25.0–45.0)	0.157^c^
Improvement rate of MUPDRS III (%)^e^	64.02 ± 0.0	65.71 ± 7.5	0.692^b^
Distance from residence to programming center (km)	100.0 (14.0–228.0)	20.0 (15.0–150.0)	0.381^c^
Residence (Wuhan/other regions)	9/18	27/20	0.046^a^

*LEDD, Levodopa Equivalent Daily Dose; MUPDRS III, Modified Unified Parkinson's Disease Rating Scale III;*

a *χ^2^ test;*

b *Independent sample t-test;*

c *Wilcoxon rank-sum test;*

d *(per-op. LEDD – LEDD at the last follow-up) / pre-op*.

LEDD * 100%;

e *(pre-op. MUPDRS III – MUPDRS III at the last follow-up) / per-op*.

MUPDRS III * 100%. MUPDRS III at the last follow-up was performed with medicine-off, stimulation-on.

### Remote Programming Contents

A total of 36 times of programming were performed for the 27 patients in the remote programming group, for which the parameters were adjusted for 35 times, including 30 times of voltage adjustment, nine times of pulse width adjustment, 4 times of frequency adjustment, 10 times of contact adjustment, and six times of unipolar/bipolar adjustment. All the patients were assessed for battery voltage and contact impedance. Permission to adjust the parameter range was increased in 15 patients. Medicines were adjusted in 16 patients. Five patients were unable to raise the voltage by themselves because of limitation on voltage authority, resulting in poor symptom control, and it only took 15 min to adjust the voltage authority and improve the patient's symptoms by remote programming.

### Adverse Events

Four (14.8%, 4/27) patients developed mild dyskinesia after programming in the remote programming group, including 3 cases of limb dyskinesia and 1 case of facial dyskinesia. Voltage was not lowered to improve the symptoms of Parkinsonism better, and dyskinesia was relieved within 1 week.

### Satisfaction Questionnaire

The questionnaire showed that 97.3% (72/74) of the patients were satisfied with the surgical effect. There was a higher tendency in a desire to accept long-term programming after DBS in the remote programming group (88.9%) than in the outpatient programming group (74.5%). In the remote programming group, 85.2% (23/27) of the patients were satisfied with the remote programming because of it being convenient and economical, and less travel. In the outpatient group, 68.1% (32/47) of the patients were satisfied with the programming at the outpatient department, while 66% (31/47) of the patients were willing to try remote programming in the future, because it was convenient and economical. However, 34% (16/47) of the outpatients were not interested in remote programming. The main reason was that residence was close to the programming center. Other reasons included difficulty of the procedure of remote programming to elderly people and complexity of patient conditions (Table 2).

**Table 2:** Satisfaction questionnaire results.

	**Question**	**Range**	**Remote programming group *n* = 27**	**Outpatient programming group *n* = 47**	**Total**	**Testing value^a^**	***P***	**Remark**
First part	Are you satisfied with the surgical effect of DBS?	Yes No	26 (96.3%) 1 (3.7%)	46(97.8%) 1 (2.1%)	72 (97.3%) 2 (2.7%)	0.242	0.623	
	Will you accept long-term programming after DBS?	Yes No	24 (88.9%) 3 (11.1%)	35(74.5%) 12 (25.5%)	59 (79.7%) 15 (20.3%)	1.598	0.206	
Second part	Remote group							
	Are you satisfied with remote programming?	Yes No	23 (85.2%) 4 (14.8%)					The reason for satisfaction: Economical; Convenient; Reducing the pain of travel; Timely solving problems
	Outpatient group							
	Are you satisfied with outpatient programming?	Yes No		32 (68.1%) 15 (31.9%)				
	Would you like to try remote programming?	Yes No		31 (66.0%) 16 (34.0%)				Reasons for willingness: Convenient, Economical. Reasons for unwillingness: The residence is located near PD Center; The procedure was difficult for the elderly; Patients' conditions were complex.

a *χ^2^ value; PD, Parkinson's disease*.

### Expenditure and Time-Cost Analysis

Thirty-six times of remote programming and 93 times of outpatient programming were performed. In the outpatient programming group, the cost of each visit was $59.5 (56–82.7), including $5.8 (2.3–29) for transportation, $10.2 for outpatient services, and $43.5 for absence from work, and was much lower in the remote programming group ($43.5 for programming, *P* < 0.001). In addition, the time cost for each programming was 30 min (25–30) in the remote programming group, while it was much longer in the outpatient programming group (150 min, 135–270, *P* < 0.001), most of which is cost of traveling (Table 3).

**Table 3:** Expenditure and time-cost for each programming in the remote and outpatient programming group.

	**Remote programming group** ***n*** **= 36**	**Outpatient programming group** ***n*** **= 93**	***P***
Programming time (min)	30.0 (25.0–30.0)	150.0 (135.0–270.0)	<0.001^a^
Total costs (US dollars)	43.5	59.5 (56.0–82.7)	<0.001^a^
Transportation fee	0	5.8 (2.3–29.0)	
Medical service fee	43.5	10.2	
Expense for absence from work	0	43.5	

a *z value*.

## Discussion

### Safety, Effectiveness, and Economical Efficiency of Remote Programming

At present, DBS is an important treatment for movement disorders and involves continuous delivery of an electrical pulse through implanted electrodes connected to an IPG, and it is programmable in amplitude, pulse width, and frequency. The adjustment of stimulation parameters requires experienced clinicians and repeated visits to achieve maximum treatment benefit, which increased the burden for patients and their families. Therefore, tele-technology for remote programming was developed to solve this problem (14, 15). This study shows that remote programming can overcome geographical barriers between doctors and patients, and provide better medical services for patients economically and timely. Functions of traditional outpatient programming, including medical history collection, physical examination (MUPDRSIII), and parameters and medication adjustments, are also available for remote programming. In addition, patients are generally satisfied with this new technique, and only four cases of mild adverse reactions occurred but were gradually alleviated. It was very difficult for the patients to travel to the outpatient clinic by public transportation because of the restriction of Parkinson's disease on motor function. Almost all the patients needed family members to drive or reserve special vehicles to visit the hospital, which led to high travel expenditure. What is more, dates of outpatient programming were on working days, which led to loss of 1 or 2-day salary for families. On the contrary, the patients in the remote programming group only needed to afford the remote programming fee.

### Satisfaction Questionnaire

The questionnaire showed that almost all the patients were satisfied with the surgical effect and long-term postoperative programming, and that remote programming had a higher satisfaction with advantages of overcoming restrictions in time and space, allowing the patients to make a programming appointment with their doctors timely and reducing the inconvenience of long-distance travel and financial pressure on the patients. Specifically, we asked the outpatients why they were not willing to try remote programming and found that most of them live near the programming center, which costs less and was relatively convenient. In general, the limitation of Parkinson's disease on patients' motor function makes all patients show a positive attitude toward a more convenient programming method.

### Remote Programming Proposals

Similar to traditional programming, remote programming also follows the standard programming principle (16). Patients often require adjustment of parameters because of gait disturbance, rigidity, tremor, or speech disorder. Increasing voltage or pulse width, changing bipolar stimulation to unipolar stimulation or single contact to dual contact stimulation can improve gait disturbance and rigidity. Poor tremor control can be improved by higher contacts stimulating the zona incerta or changing the single contact to double contact stimulation. Reducing pulse width or higher contact is helpful to speech disorder. In addition, medication can be properly adjusted according to patients' conditions. Attention should be paid to the following matters in remote programming: first, doctors should know each patient's stimulation targets, electrode depth and position, and main demands for programming; second, a wide range of adjustments, such as changing contacts or bipolar stimulation to unipolar stimulation, should be carefully carried out; third, when clinicians adjust the parameters, patients should sit safely to prevent falls; fourth, the authority of the patient programmer should be properly set within a safe range.

### Dyskinesia

Because of inappropriate stimulation during programming, patients may experience symptoms, such as dysphonia, dizziness, and dyskinesia. For outpatients, we can observe and adjust timely, so adverse reactions during programming can be eliminated in the clinic. This procedure could not be carried out during remote programming limited by time and space. Therefore, in this study, we did not collect adverse reactions in the outpatient programming group. Dyskinesia was the only adverse reaction in the remote programming group, and was mainly manifested as involuntary movement and stereotype of limbs or the body after adjusting stimulation parameters or/and taking levodopa. The mechanism of dyskinesia is not completely clear. Studies suggest that dyskinesia is related to the long-term use of levodopa, and that about 40% of patients developed dyskinesia after 4 years of levodopa treatment (17–19). Although the dosage of the drug was significantly reduced after DBS, some patients may develop dyskinesia under the superposition of drugs and stimulation, especially for patients with preoperative drug-induced dyskinesia. Usually, stimulus-induced dyskinesia will gradually weaken or disappear after a few days or months. In addition, dyskinesia can be alleviated or eliminated by changing the intensity of the stimulus, adjusting stimulation contact, choosing bipolar stimulation mode, or reducing the dose of dopaminergic drugs and changing the timing of medication.

### Shortcoming of Remote Programming

Remote programming is not flawless. Clinicians cannot directly perform physical examinations on patients through video communication, which makes clinicians unable to know the patients' conditions very well. Therefore, remote programming cannot solve all problems for patients with complex conditions. Both doctors and patients should have a reasonable expectation on remote programming. If the patient's physical signs are transmitted in real-time in combination with wearable devices, it can partially make up for the lack of physical examination (20). In addition, equipment and network conditions can also affect the smooth progress of programming. Nevertheless, remote programming still has incomparable advantages over traditional programming and has broad application prospects. Especially, in the context of the coronavirus disease 2019 (COVID-19) pandemic, remote programming has become the ideal method for programming in patients with PD after DBS. With the application and popularization of the fifth-generation mobile communication technology (5G), remote programming will be further improved and will play an increasingly important role in postoperative programming for patients with PD.

## Conclusion

Remote programming is safe and effective after DBS in patients with PD. Moreover, it reduces expenditure and time costs for patients and achieves high satisfaction, particularly for patients living far from programming centers.

## Data Availability Statement

The raw data supporting the conclusions of this article will be made available by the authors, without undue reservation.

## Ethics Statement

The studies involving human participants were reviewed and approved by the Medical Ethics Committee of Zhongnan Hospital of Wuhan University. The patients/participants provided their written informed consent to participate in this study.

## Author Contributions

PN and JieZ contributed to the conception and design of the study. XY, YS, XZ, WL, and KF contributed to the acquisition and analysis of the data. PN, JibZ, and JieZ contributed to the drafting of the manuscript. All authors contributed to the article and approved the submitted version.

## Funding

This study was supported by the National Key Development Plan of China (grant no. 2016YFC0105900; responsible for the study organization and conduct).

## Conflict of Interest

The authors declare that the research was conducted in the absence of any commercial or financial relationships that could be construed as a potential conflict of interest.

## Publisher's Note

All claims expressed in this article are solely those of the authors and do not necessarily represent those of their affiliated organizations, or those of the publisher, the editors and the reviewers. Any product that may be evaluated in this article, or claim that may be made by its manufacturer, is not guaranteed or endorsed by the publisher.
